# MetaboLab - advanced NMR data processing and analysis for metabolomics

**DOI:** 10.1186/1471-2105-12-366

**Published:** 2011-09-13

**Authors:** Christian Ludwig, Ulrich L Günther

**Affiliations:** 1 The Henry Wellcome Building for Biomolecular NMR Spectroscopy, School of Cancer Sciences, University of Birmingham,Edgbaston, Birmingham B15 2TT, UK

## Abstract

**Background:**

Despite wide-spread use of Nuclear Magnetic Resonance (NMR) in metabolomics for the analysis of biological samples there is a lack of graphically driven, publicly available software to process large one and two-dimensional NMR data sets for statistical analysis.

**Results:**

Here we present MetaboLab, a MATLAB based software package that facilitates NMR data processing by providing automated algorithms for processing series of spectra in a reproducible fashion. A graphical user interface provides easy access to all steps of data processing via a script builder to generate MATLAB scripts, providing an option to alter code manually. The analysis of two-dimensional spectra (^1^H,^13^C-HSQC spectra) is facilitated by the use of a spectral library derived from publicly available databases which can be extended readily. The software allows to display specific metabolites in small regions of interest where signals can be picked. To facilitate the analysis of series of two-dimensional spectra, different spectra can be overlaid and assignments can be transferred between spectra. The software includes mechanisms to account for overlapping signals by highlighting neighboring and ambiguous assignments.

**Conclusions:**

The MetaboLab software is an integrated software package for NMR data processing and analysis, closely linked to the previously developed NMRLab software. It includes tools for batch processing and gives access to a wealth of algorithms available in the MATLAB framework. Algorithms within MetaboLab help to optimize the flow of metabolomics data preparation for statistical analysis. The combination of an intuitive graphical user interface along with advanced data processing algorithms facilitates the use of MetaboLab in a broader metabolomics context.

## Background

### One-dimensional NMR

Metabolomics has become an important technique in the context of systems biology to characterize changes in metabolite composition and concentration in biological systems such as cells, tissues or in bio-fluids. One-dimensional (1D) NMR spectra used in the context of metabolomics contain hundreds of signals arising from 50-100 metabolites. To utilize this wealth of information in the context of statistical analysis, consistent and accurate processing of the data is paramount. This includes phase correction of complex NMR signals to pure absorption line shapes and consistent baseline correction across series of spectra, along with various linear and non-linear scaling algorithms and spectral alignment (see additional file for more information). Scaling includes linear scaling algorithms, specifically total spectral area scaling and probabilistic quotient normalization [1]. Among non-linear scaling algorithms it includes the generalized logarithmic transformation (glog), Pareto or autoscaling [2], used prior to statistical processing. Excellent reproducibility within series of spectra is essential for subsequent statistical analysis using multivariate algorithms such as principal component analysis (PCA) or PLS-DA, but also for univariate analysis and signal integration.

To become accessible to a broader range of users in a translational setting, metabolomics software needs to provide intuitive and transparent control over all processing steps, without limiting more sophisticated uses. This need has been addressed by a batch processing interface, suitable to handle larger series of spectra with standard processing parameters, with an option to create user editable scripts allowing more sophisticated changes and providing access to algorithms from other packages.

### Two-dimensional NMR spectra

The fundamental requirements for spectral processing of two-dimensional (2D) NMR spectra, such as *J*-resolved or HSQC spectra, are similar to those for 1D-NMR spectra. Owing to the reduced congestion in 2D spectra and the smaller number of data points, algorithms such as baseline correction are easier to implement. 2D spectra also present additional opportunities, especially for the assignment of metabolites depending on user driven decisions for ambiguous signals. This requires a spectral library, ideally one that has been recorded for the same field strength and sample conditions (e.g. solvent composition, buffer, sample pH and temperature). MetaboLab implements an easy mechanism to add spectral data for metabolites taken from databases.

Assignment decisions will often depend on the size of signal intensities in relation to other signals of the same metabolite, facilitated by the display of intensity values for picked peaks within regions of interests for the resonances of the metabolite of interest. MetaboLab also includes a simple mechanism to transfer peak lists between spectra and automatically adjusts small shifts of signals arising from pH variations.

## Results and Discussion

### Objectives

MetaboLab is based on the previously developed NMRLab software [3], which is a general purpose software tool for multidimensional NMR data processing similar to other freely available software [4-6]. MetaboLab aims to facilitate post-processing steps necessary to prepare NMR spectra for statistical analysis similar to the script based software ProMetab [7]. The major objectives for the MetaboLab software design were,

1. to create a simple and transparent software interface providing access to state-of-the-art algorithms,

2. to provide automated or graphically facilitated algorithms, e.g. for phase correction, spectral referencing, glog transform optimisation, baseline correction of series of spectra,

3. to create a graphical batch processing interface,

4. to enable data output for statistical packages such as PLS Toolbox (Eigenvector Research Inc.),

5. to enable semi-automated assignment of 2D-HSQC spectra,

6. to provide tools capable of generating peak lists and intensities for series of spectra.

### One-dimensional NMR spectra processing and batch processing

MetaboLab includes the processing of the original spectrometer data (implemented for Bruker and Varian/Agilent data and parameter files), followed by common processing steps. The free induction decay (FID) is Fourier transformed, phase corrected and referenced, a robust automated algorithm is included for phase correction, which has worked well for most spectra acquired with the Bruker 'baseopt' acquisition parameter. Automated referencing to chemical shift standards such as TMS, TMSP or DSS is available. If no chemical shift standard has been added referencing is performed on the assumption that the transmitter offset has been set to the water frequency [8].

For batch processing of NMR series of NMR spectra a script builder generates scripts using standard processing steps including zero filling, several algorithms for post acquisition water suppression [9], apodization functions, phase correction (two different automated algorithms or manual phase correction), DC offset correction, Gibbs multiplication and referencing. The file selection mechanism allows the simultaneous choice of NMR data from different directories. The script builder composes a MATLAB script, which can be further edited and executed. This script will read all the selected FIDs and process them subsequently (see also Additional File 1).

Processing scripts can similarly be composed for 2D spectra. Parameter sets for 2D *J*-resolved and for HSQC spectra have been included. For a series of 2D *J*-resolved spectra a projection using either the skyline or summation algorithm along the frequency axis can be applied after optionally calculating a tilted 2D spectrum. Apodization functions default to either exponential multiplication for 1D spectra or square cosine for 2D spectra. A window function composed of a shifted sine multiplied by an exponential (SEM window function [10]) is also available for 2D *J*-resolved spectra.

### MetaboLab: Metabolomics post-processing

**Figure 1:**
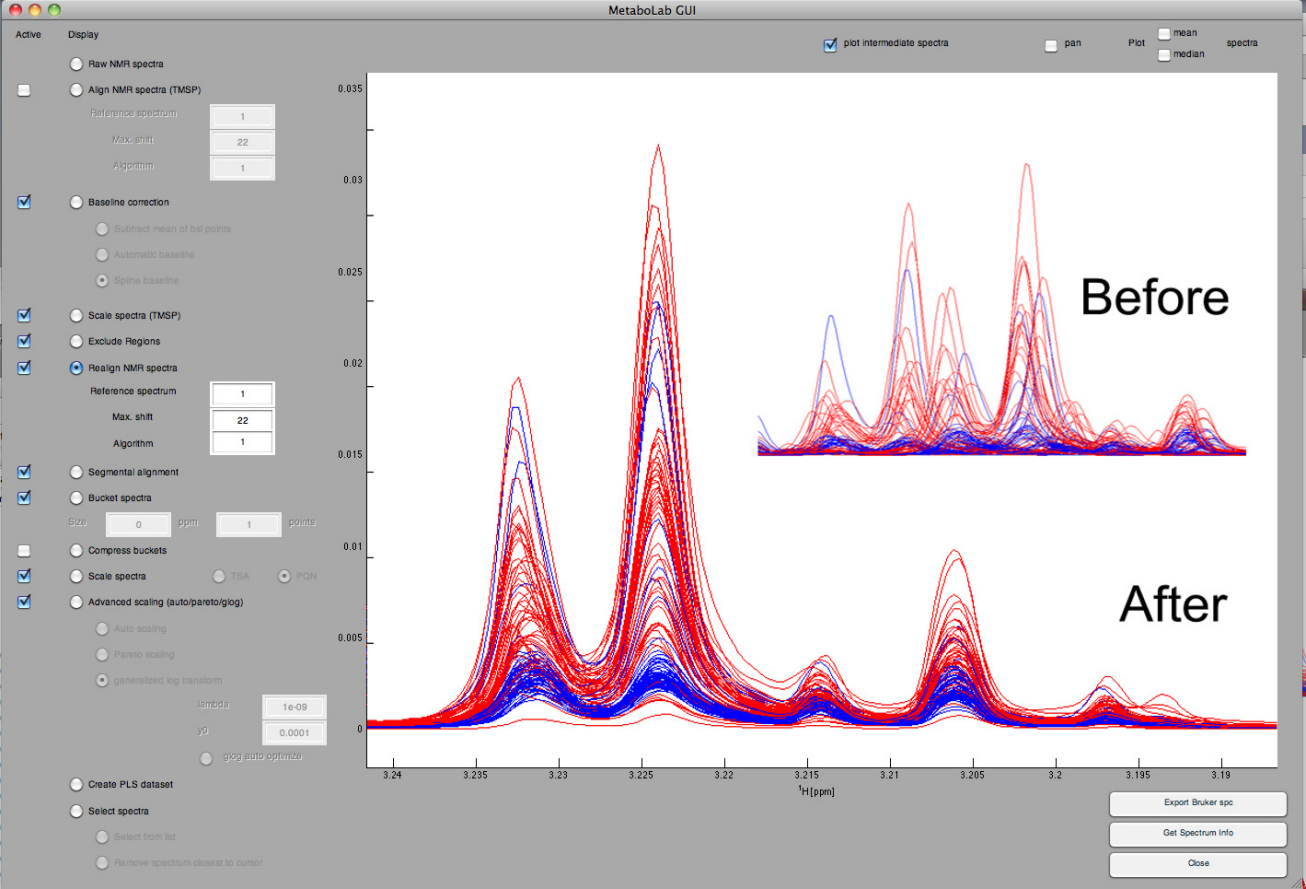
**1D-NMR spectra**. MetaboLab graphical user interface showing an overlay of 329 1D-NMR spectra of extracted tissue biopsies before (large spectra) and after post-processing (insert spectra). Spectra from cancerous tissue are plotted in blue while those originating from control samples are shown in red. The NMR spectra were aligned to TMSP, baseline corrected and the water region and regions without peaks were excluded. The spectra were then scaled to the total spectral area and re-aligned with respect to the first spectrum.

Metabolomics NMR data analysis requires additional post-processing applied to the data series in order to remove distortions causing artifacts in subsequent statistical data analysis or quantification. Post-processing includes spectral alignment (with respect to TMSP or a reference spectrum, the latter is typically performed after selected regions of the spectra have been excluded), various algorithms for baseline correction, the scaling of spectra (with respect to TMSP or total spectral area), bucketing and data export for subsequent multivariate statistical analysis. The graphical user interface (Figure 1) allows for the exclusion of spectra and the assignment of classes, which can be displayed in different colors. An interface is provided to transfer data and spectral classes into the commercially available PLS Toolbox software (Eigenvector Research Inc., Wenatchee, USA).

The MetaboLab graphical user interface provides easy access to post-processing tasks. Even on modern spectrometers the spectral baseline is often not ideally flat or shows a small DC offset. Such distortions typically may arise from the use of digital filters in the context of oversampling or from imperfections in the water suppression. Moreover, small baseline distortions in high-resolution 1D spectra are enhanced by non-linear scaling algorithms such as the glog transform [2], Pareto or autoscaling, which are typically used to enhance small signals in spectra. MetaboLab includes various algorithms with automated determination of baseline points for baseline correction [11,12]. However, parametrization of these algorithms is often difficult and tends to cause artifacts at the edge of peaks. Best results are achieved by manually picking baseline points on the series of NMR spectra, using common zeros in all or most spectra, followed by calculating a spline function to model the baseline for each individual spectrum. The advantage of this is that exactly the same spline points can be used for all spectra, thus reducing the variability between spectra. Facilitated by a graphical interface this helps to minimize distortions introduced by the baseline correction.

MetaboLab includes a new algorithm to compute an optimized glog parameter that maximizes the variance in the spectral series of NMR spectra. For sufficiently large data sets this algorithm yields good glog parameters. MetaboLab also allows manual modification of glog parameters and facilitates its adjustment by updating the display of processed spectra in real time.

### Graphical Assignment Tool

**Figure 2:**
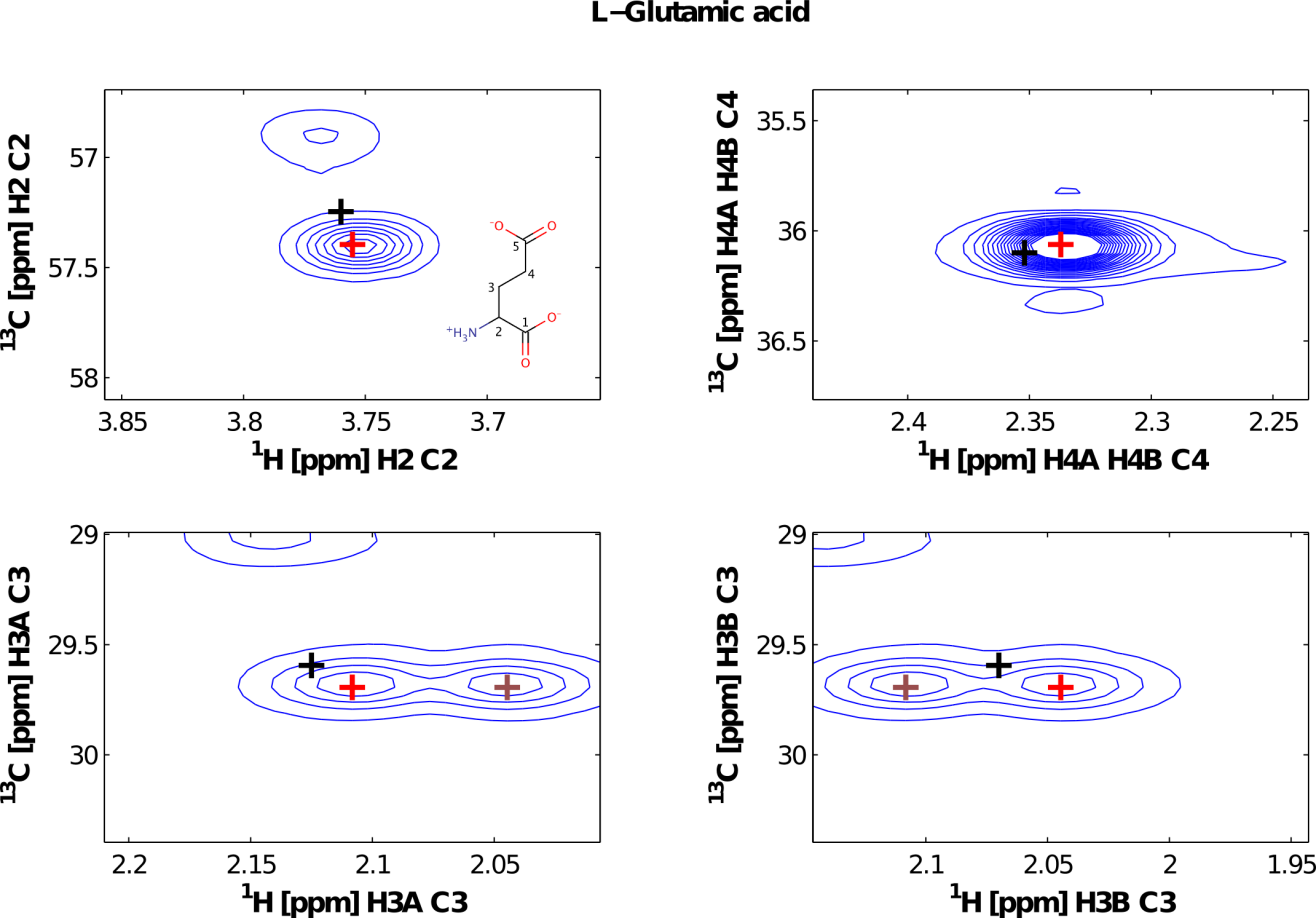
**HSQC spectra**. The MetaboLab assignment tool showing sections of a HSQC spectrum for the resonances of glutamate. The library chemical shift is marked with a black '+' whereas the actual picked peaks are displayed in red. A picked peak of another nucleus in the same molecule in the displayed regions is shown in dark red. The molecular structure depicts the nomenclature for each nucleus.

The assignment tool presents itself as a graphical user interface to assign signals in 2D-HSQC spectra based on metabolite peak lists in a spectral library (Figure 2). MetaboLab contains a limited number of assigned metabolites from public databases [13] but provides an easy method to add additional metabolites. The software allows for small differences in chemical shifts in a spectrum compared to those in the library and searches for the closest local maximum (similar to the region of interest based data analysis in the rNMR program [14]). The chemical structure including atom numbering of the carbon nuclei is displayed for an individual metabolite (see Additional File 1 for details). For ambiguous assignments alternative assignments available in the spectral library can be displayed along with peak intensities. Duplicate assignments are highlighted by color.

## Conclusions

MetaboLab has been designed to provide an intuitive user-friendly software for metabolomics data processing and analysis, which is equally usable for non-expert users as for users interested in further data analysis within MATLAB. The software is designed to process series of NMR spectra in the most reproducible manner. The software can be used via its graphical user interface but providing access to all data structures from within the MATLAB environment. All functions in MetaboLab/NMRLab are scriptable and scripts can be generated by a graphical front-end. Baseline correction, phase correction, alignment and scaling tools were designed for optimal processing of larger series of spectra. MetaboLab also provides a link to the MATLAB based statistical data analysis software PLS toolbox.

The software can be downloaded from http://www.nmrlab.org.uk. This website also contains online manuals and tutorial videos.

## Methods

The software tools described in this article were implemented within the MATLAB programming environment, using algorithms from the pre-existing NMRLab software package [3]. Additional tools and algorithms that are designed for metabolomics data analysis and metabolic flux analysis include a baseline correction algorithm based on spline functions to model the spectral baseline and an advanced automatic phase correction algorithm for 1D ^1^H-NMR spectra.

### Automated phase correction

The automatic phase correction algorithm uses the TMSP signal if present, employing baseline properties between the TMSP signal and its ^13^C-satellites. It also uses baseline properties at the other end of the spectrum to calculate a first order phase correction. If no TMSP is present it uses baseline properties at both ends of the spectrum to determine a zero order phase correction. *Baseline correction*. The data structure used in MetaboLab reflects closely that of NMRLab [3]. It uses a MATLAB data structure (metaboSpc) with fields storing the information for the different data post-processing steps, e.g. metaboSpc.baseline contains further data structure fields for the different baseline correction algorithms. For example, metaboSpc.baseline.spline contains the field baseline_points as a vector, the field baseline_average_points as a number indicating how many baseline points are averaged around each baseline point (selected in baseline points), the field linear determining whether a linear interpolation of adjacent baseline points is to be used for regions where no spectral baseline is available within a range of linear_points. All post-processing steps can be reconstructed from the information stored in the metaboSpc data structure.

### Samples

1D-^1^H NMR spectra shown in Figures 1 were acquired from ultra-filtrated blood plasma samples [15]. NOESY-presat was used to suppress the solvent resonance. All spectra were automatically processed, phase corrected and referenced using the script builder interface before data post-processing was performed using the Metabolab GUI software. 2D-HSQC spectra were acquired from MeOH/CHCl_3 _cell extracts of K562 CML cells fed with ^13^C(1,2)-labeled Glucose, as described in [16] except for the addition of labelled glucose. *NMR spectra*. HSQC spectra were recorded using echo/anti-echo coherence selection for quadrature detection in the indirect dimension. The first spectrum was manually processed using the NMRLab software, then all spectra were automatically processed with the script builder interface using referencing and phasing information from the first spectrum. Resonance assignment was performed inside the HSQC assignment tool.

## List of abbreviations used

1D: one dimensional; 2D: two dimensional; CHCl_3_: Chloroform; DSS: Dimethylsilapentanesulfonate; FID: free induction decay; glog: generalised logarithm transformation; HSQC: heteronuclear single quantum coherence; MeOH: Methanol; NMR: nuclear magnetic resonance; NOESY: Nuclear Overhauser spectroscopy; PCA: principal component analysis; PLS-DA: partial least squares discriminant analysis; presat: presaturation of the solvent resonance; SEM: Sine and exponential multiplication; TMS: Tetrasilylmethane; TMSP: Sodium Trimethylsilyl-propionate.

## Authors' contributions

CL conceived and implemented the graphical user interfaces and the script builder application. Algorithms were conceived and implemented by UG and CL, who also wrote the manuscript. Both authors read and approved the manuscript.

## Supplementary Material

Additional File 1 **contains additional screenshots of the MetaboLab graphical user interface to illustrate several steps of data post-processing, an illustration of the user interface for the script builder application, and shows the usage of the graphical HSQC assignment tool**. Furthermore, step by step instructions are given to extend the HSQC library for the graphical HSQC assignment tool. Click here for file
