# Supplementary material for: MetaboLab - advanced NMR data processing and analysis for metabolomics
Source: BMC Bioinformatics. 2011 Sep 13;12:366. doi: 10.1186/1471-2105-12-366 (PMC3179975; doi:10.1186/1471-2105-12-366)
Supplement: Additional File 1 — contains additional screenshots of the MetaboLab graphical user interface to illustrate several steps of data post-processing, an illustration of the user interface for the script builder application, and shows the usage of the graphical HSQC assignment tool. Furthermore, step by step instructions are given to extend the HSQC library for the graphical HSQC assignment tool. [file 1471-2105-12-366-S1.PDF]

MetaboLab - advanced NMR data processing and analysis for metabolomics

Christian Ludwig, Ulrich Günther

September 8, 2011

Additional File 1

MetaboLabGUI - Class Assignment

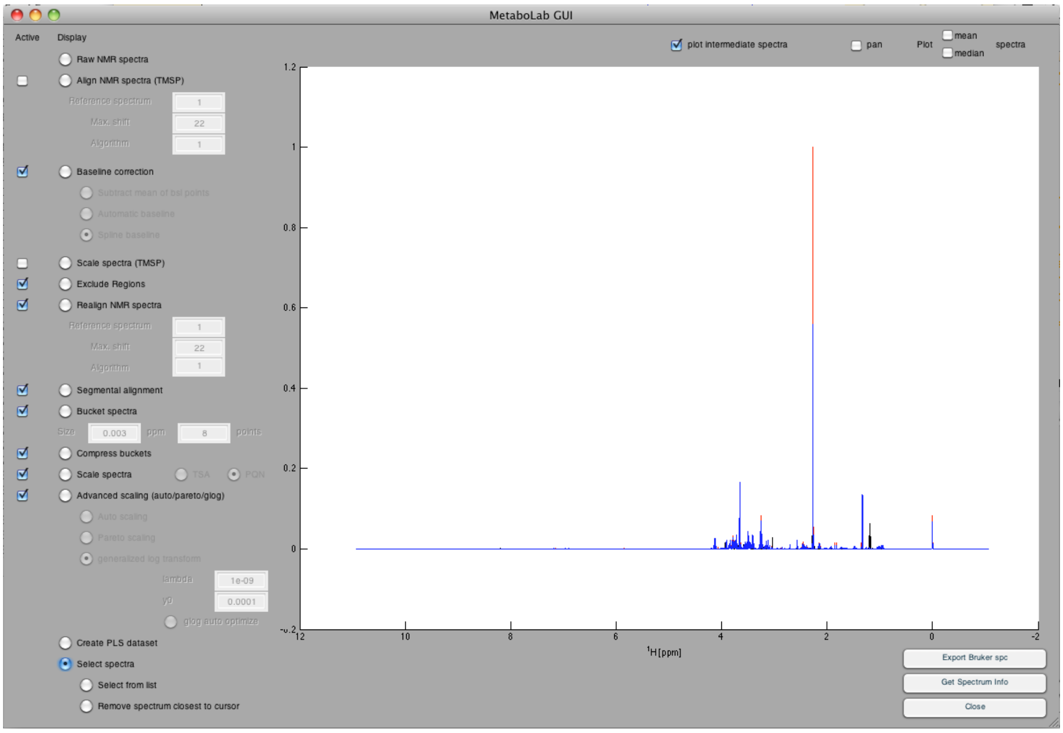

Figure 1: Initial plot of selected NMR spectra after opening MetaboLabGUI.

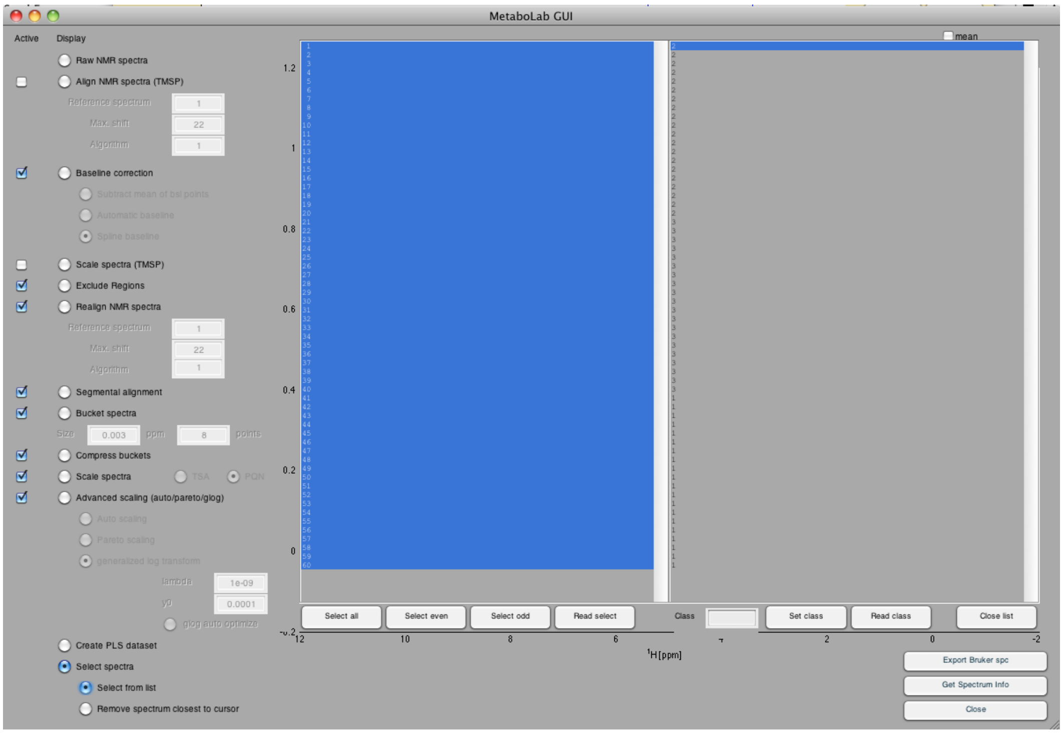

Figure 2: Spectra selection and class assignment in MetaboLabGUI.

MetaboLabGUI - Mean Plot of Spectra Classes

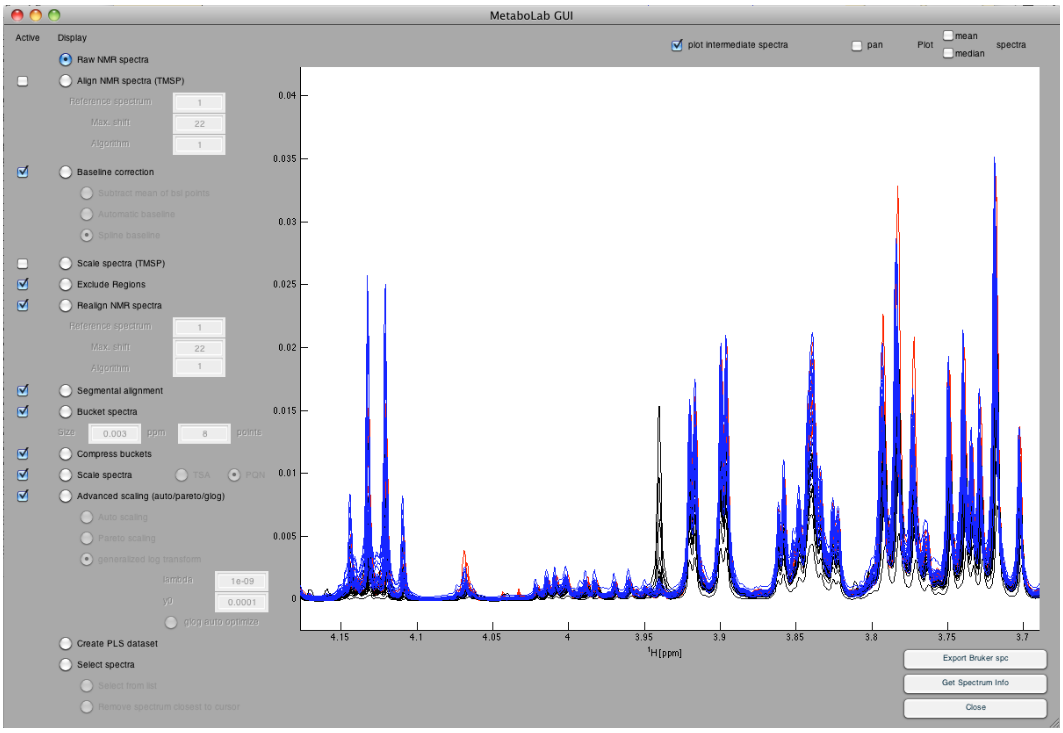

Figure 3: Expanded region of the spectra showing an overlay plot of all selected NMR spectra.

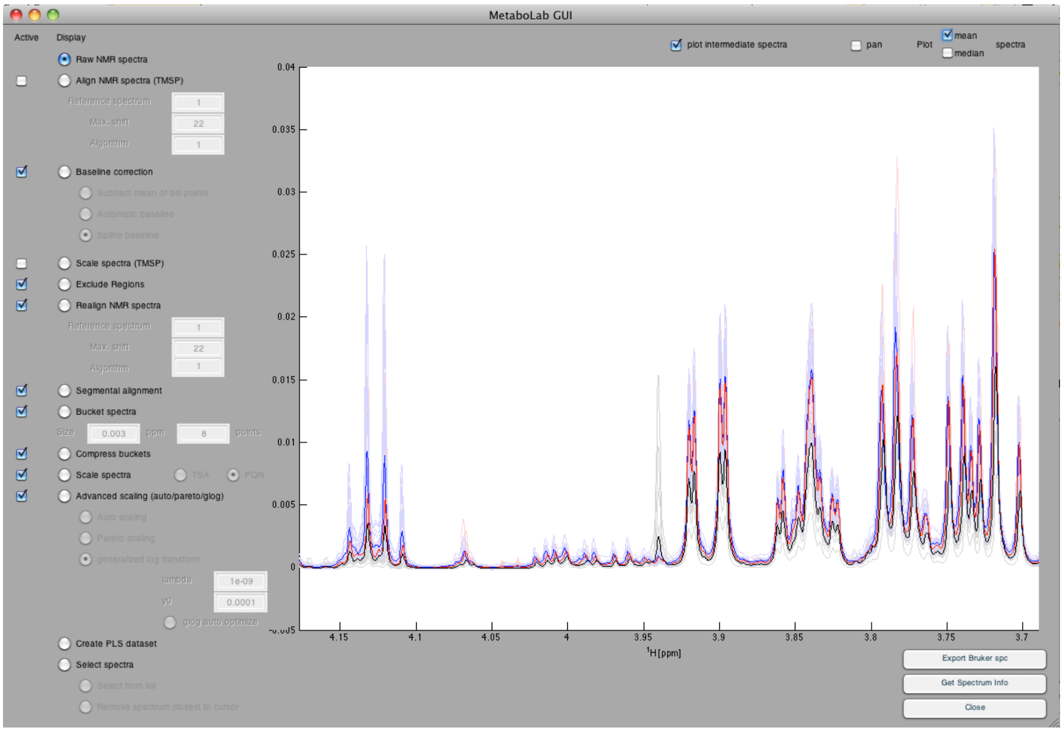

Figure 4: Expanded region of the spectra showing an overlay plot of all selected NMR spectra (faint colours) and a plot of the mean spectra of the different classes.

MetaboLabGUI - Baseline Correction

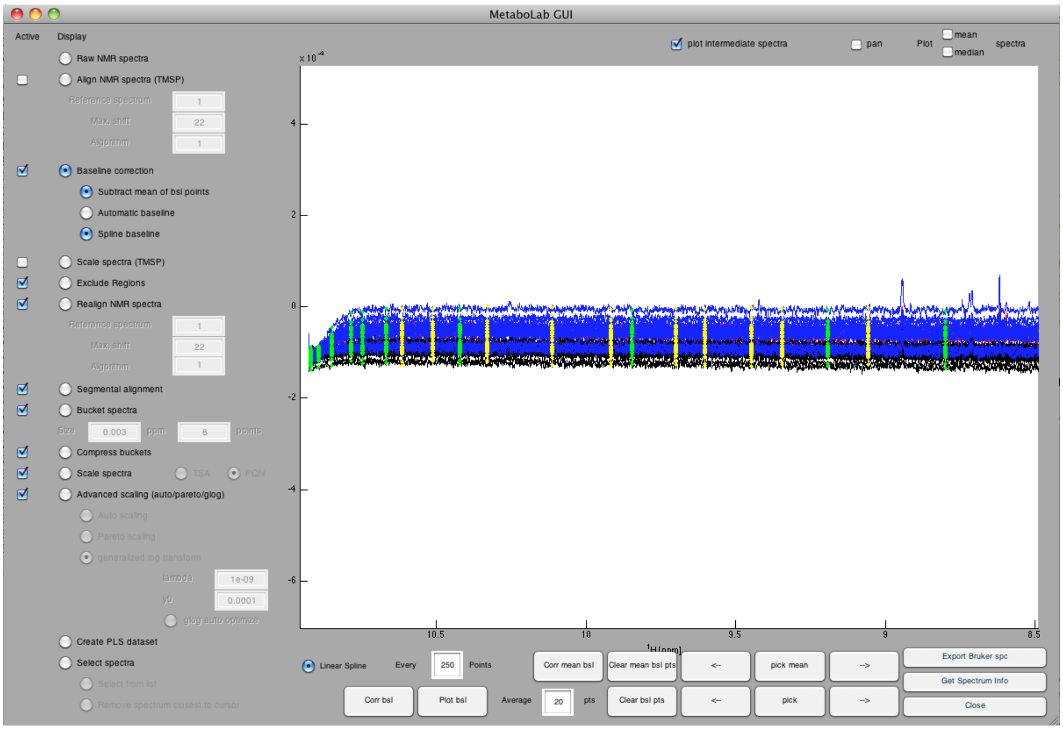

Figure 5: The left baseline region of the NMR spectra is shown. Two different methods for baseline correction are applied. First the mean value of selected baseline points is subtracted from each spectrum (yellow points). Then a spline baseline function selected by the green baseline points is subtracted from each spectrum. The results are shown in figures 8 and 9.

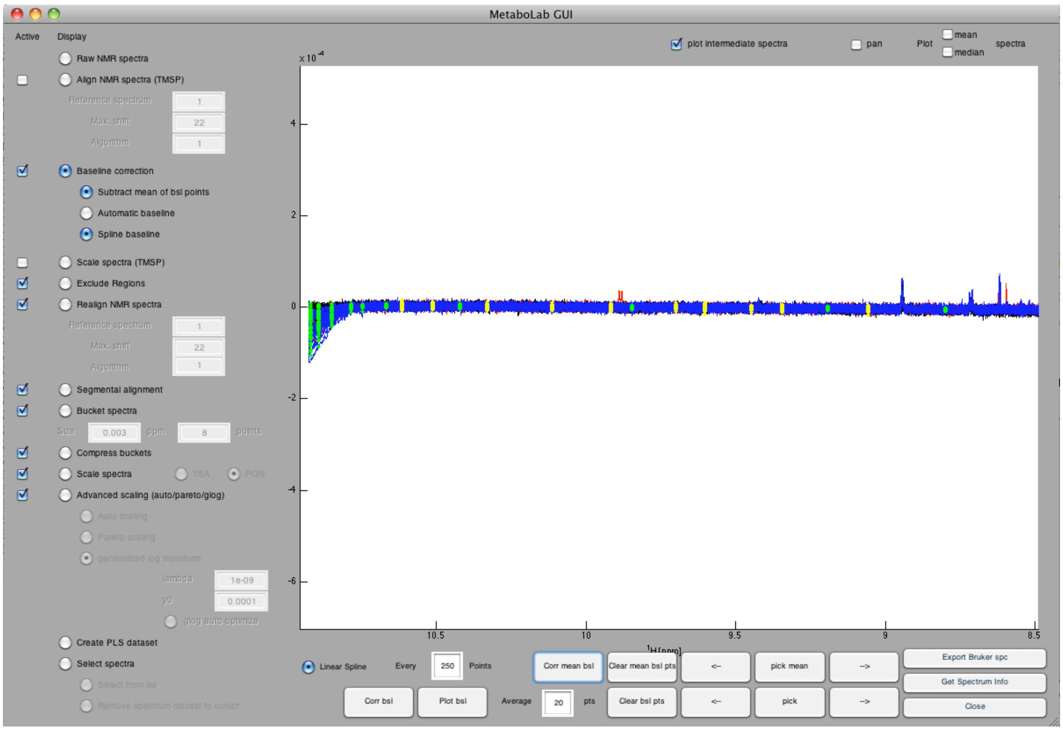

Figure 6: Same baseline region of the NMR spectra in figure 7 after performing the mean baseline correction.

MetaboLabGUI - Baseline Correction

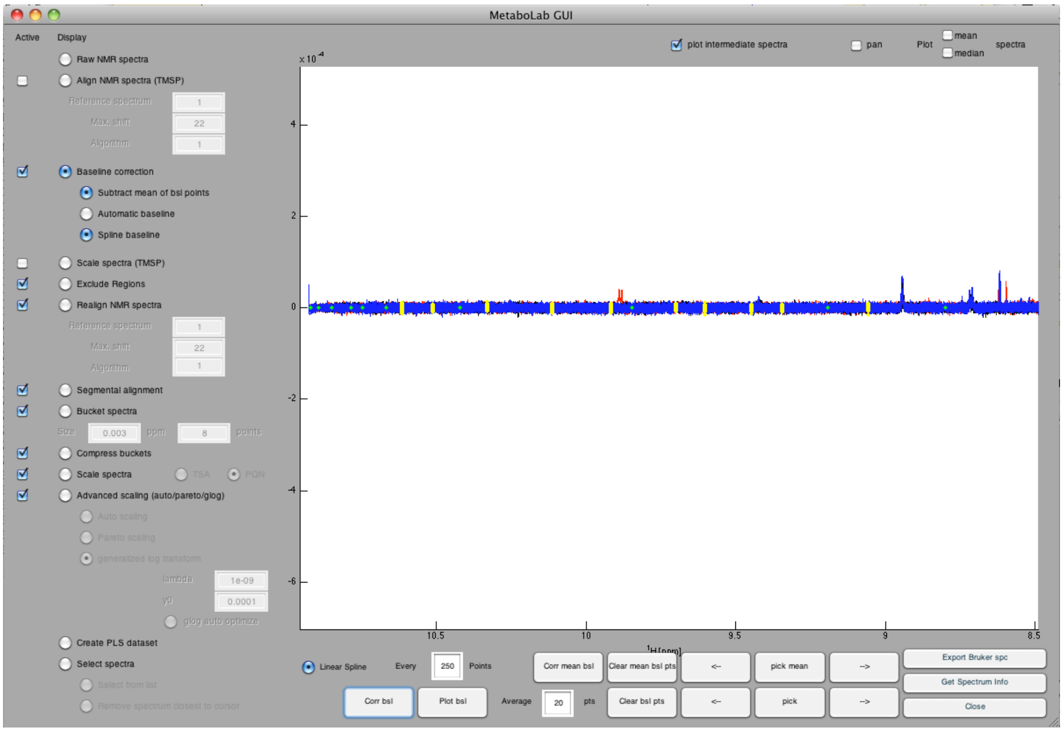

Figure 7: Baseline region of the NMR spectra after the additional spline baseline correction.

MetaboLabGUI - Baseline Correction

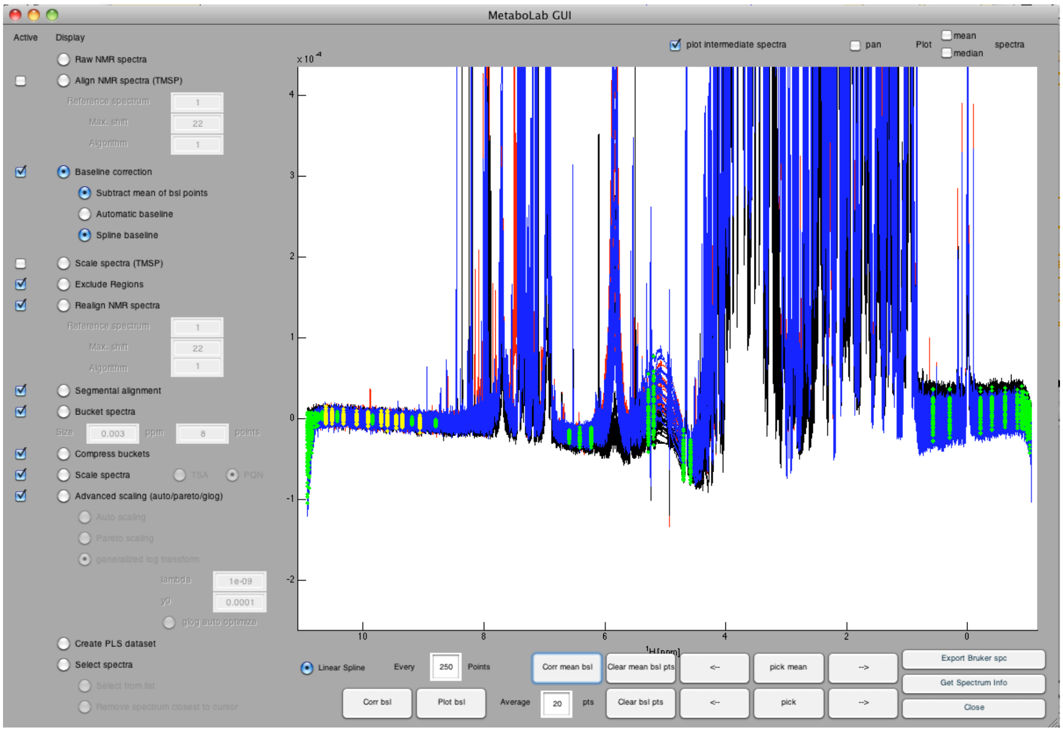

Figure 8: Baseline of the NMR spectra after mean and before spline baseline correction.

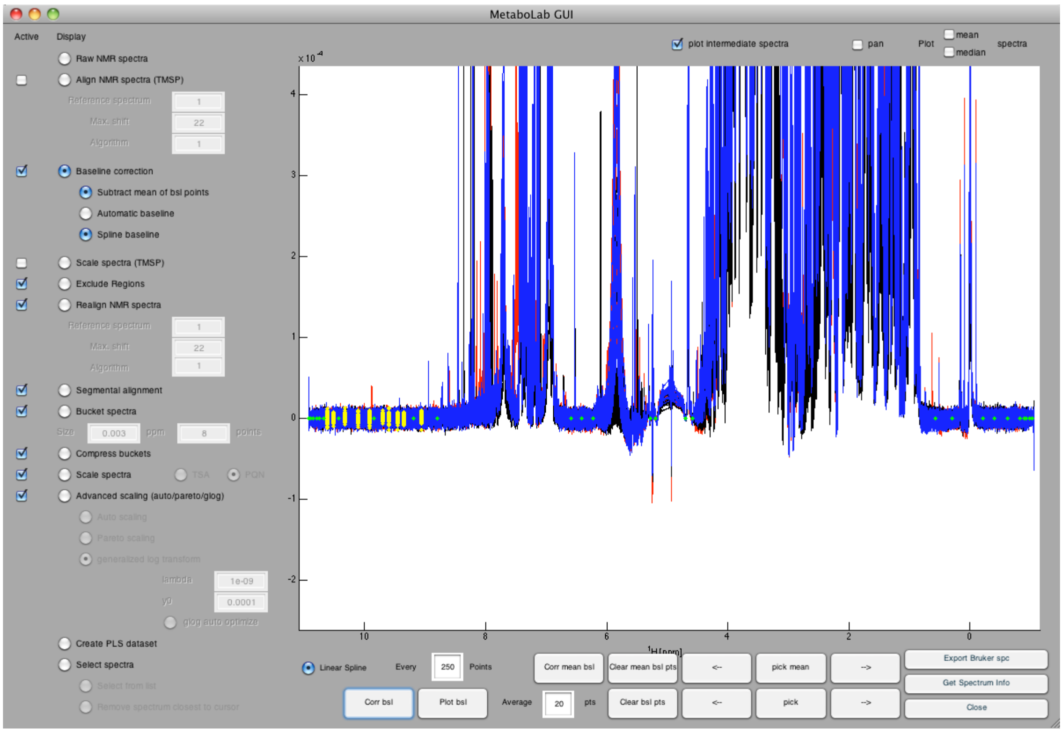

Figure 9: Baseline of the NMR spectra after mean and spline baseline correction.

MetaboLabGUI - Selection of Exclusion Regions

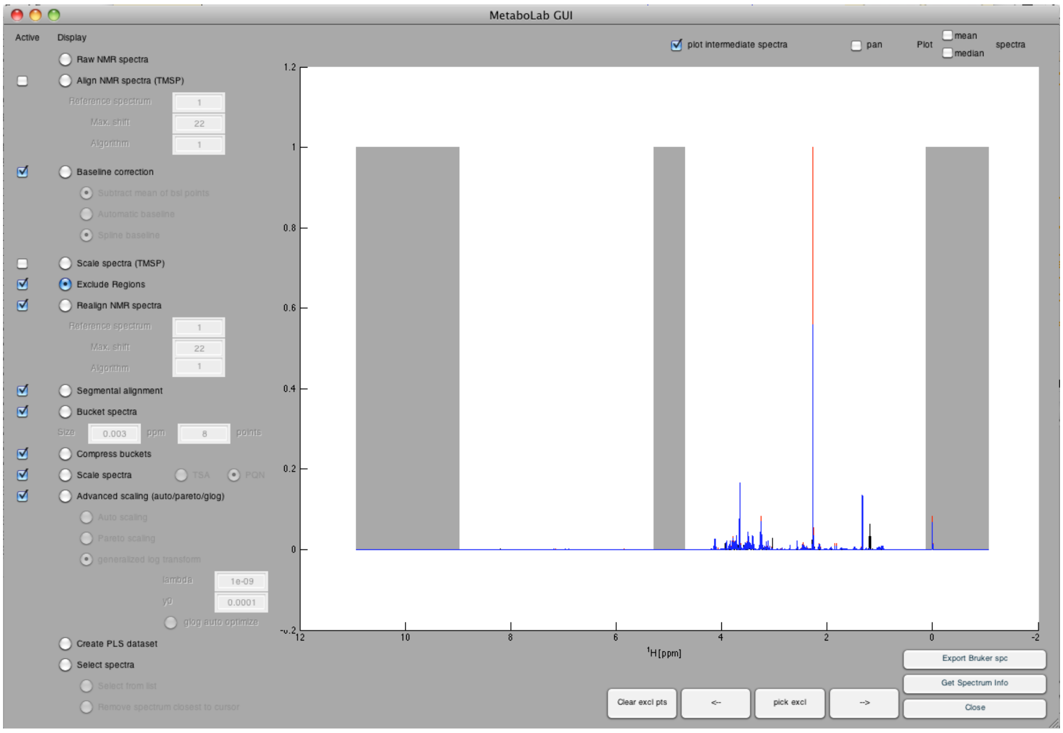

Figure 10: Selection of regions to be excluded from statistical data analysis (e.g. water region and baseline regions at the edges of the spectra).

MetaboLabGUI - Advanced Scaling

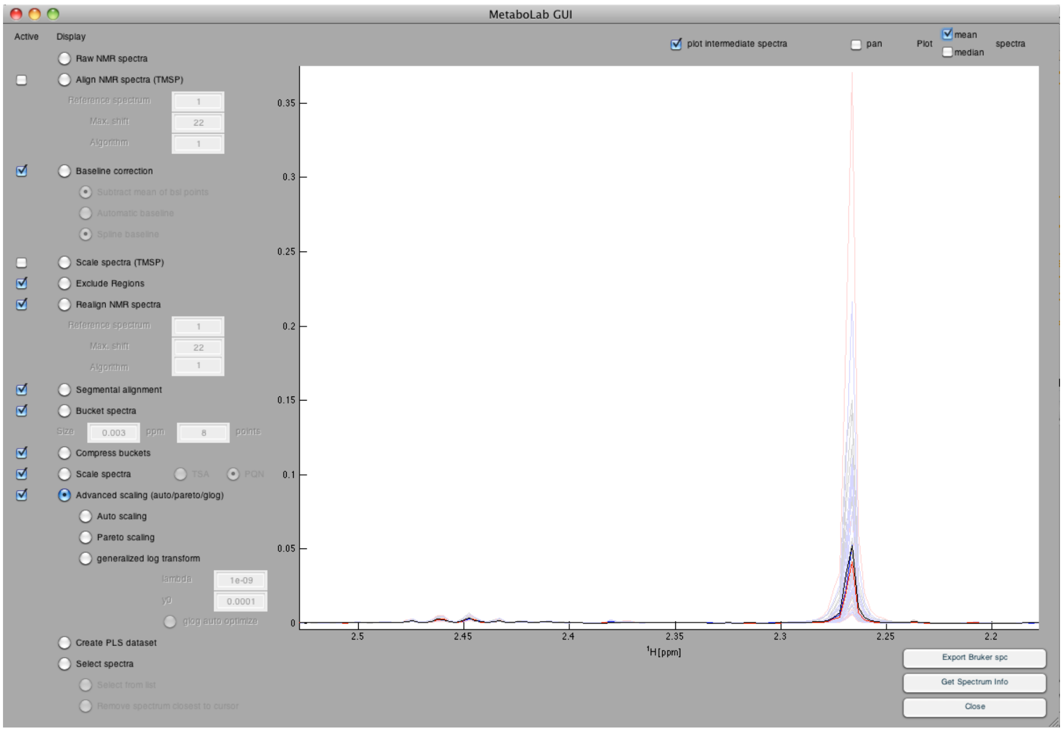

Figure 11: Expanded region of NMR spectra before advanced scaling (mean plot).

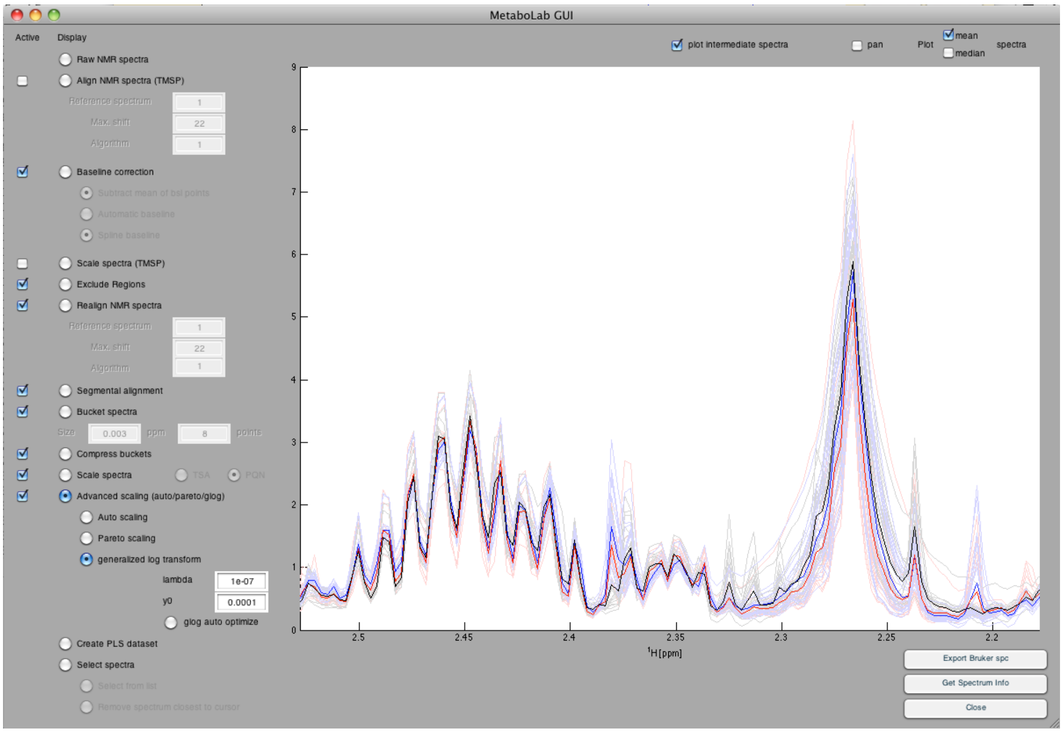

Figure 12: Expanded region of NMR spectra after applying a glog transform, showing the mean spectra.

ScriptBuilder Interface

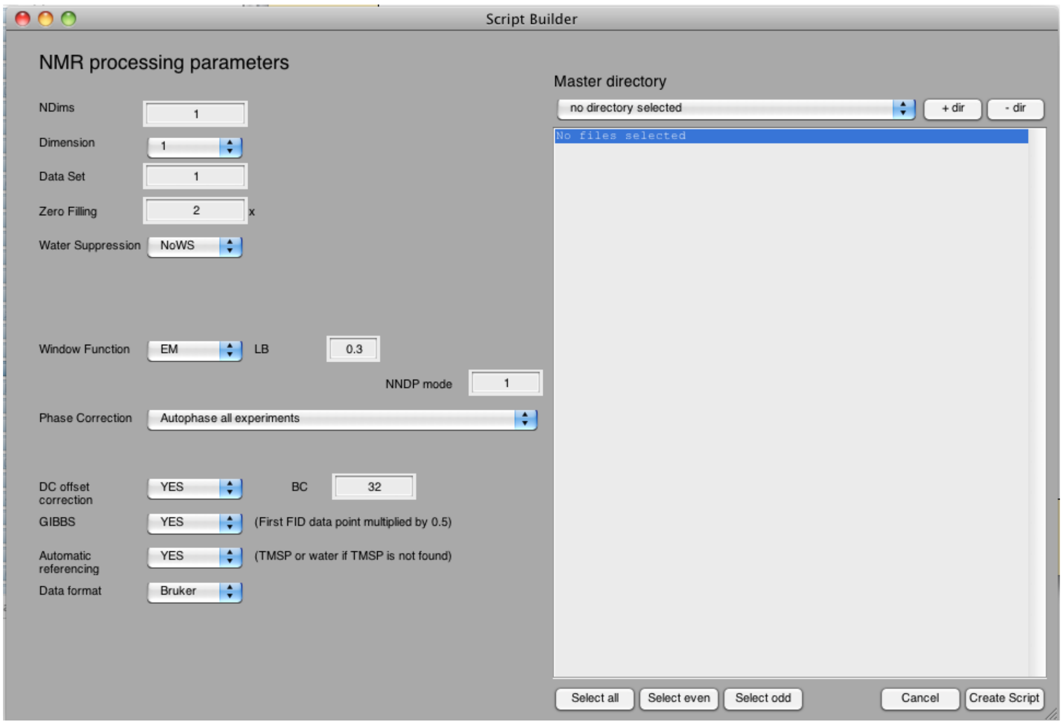

Figure 13: The script builder user interface: Processing parameters are set in the left part of the figure, directories with data selection on the right.

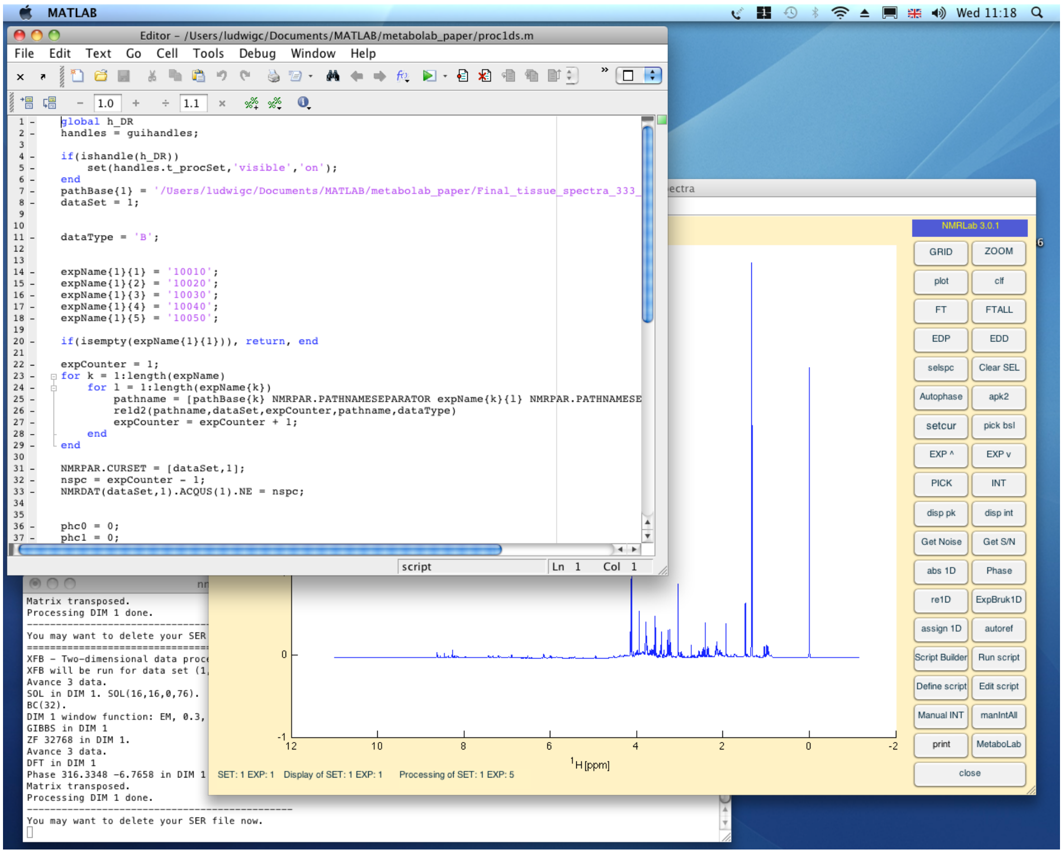

Figure 14: MATLAB script automatically generated by the script builder interface. The first processed NMR spectrum of the series is shown in the background.

HSQC assignment tool

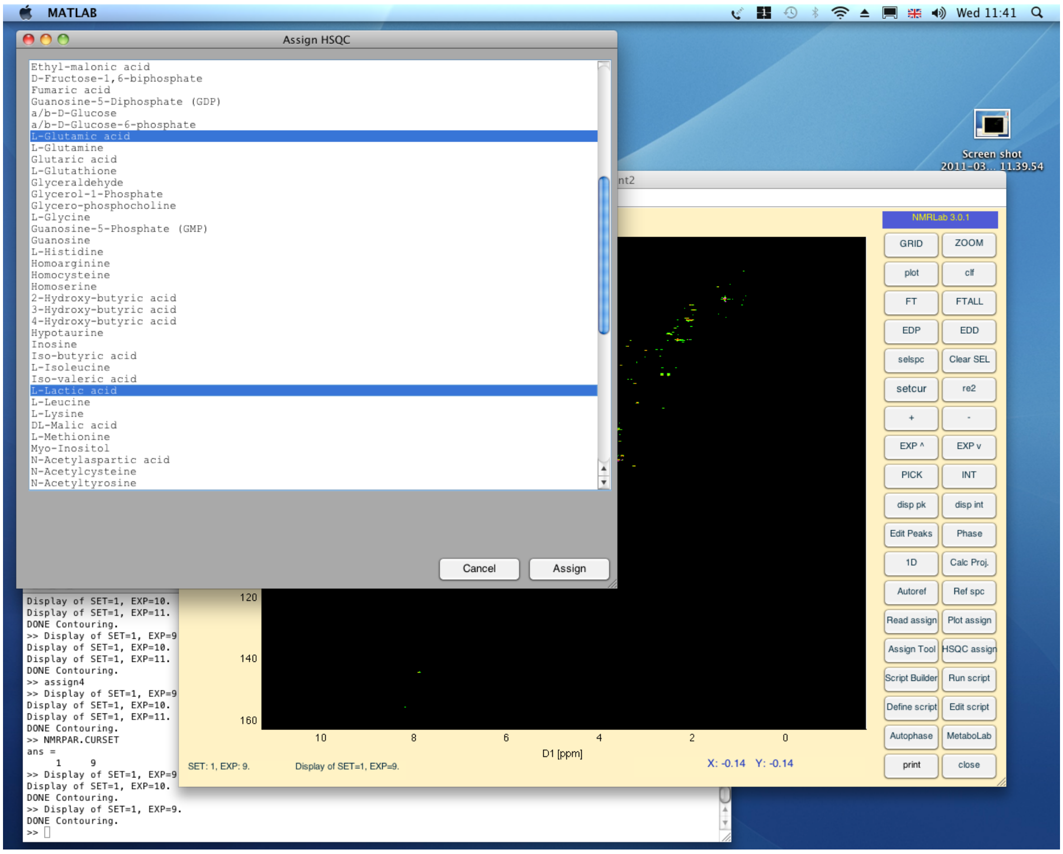

Figure 15: Basic HSQC assignment tool with selection dialog to select metabolites from the spectral library for assignment.

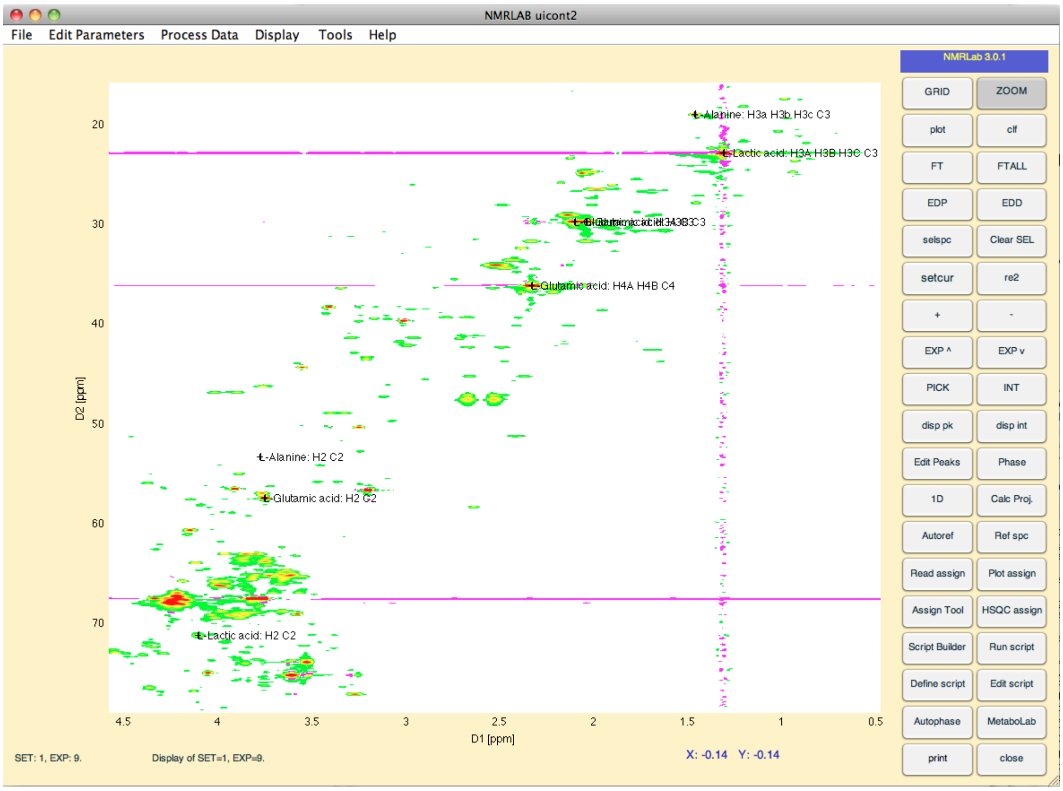

Figure 16: 2D-^{13}C,^{1}H-HSQC spectrum with assigned resonances.

HSQC assignment tool

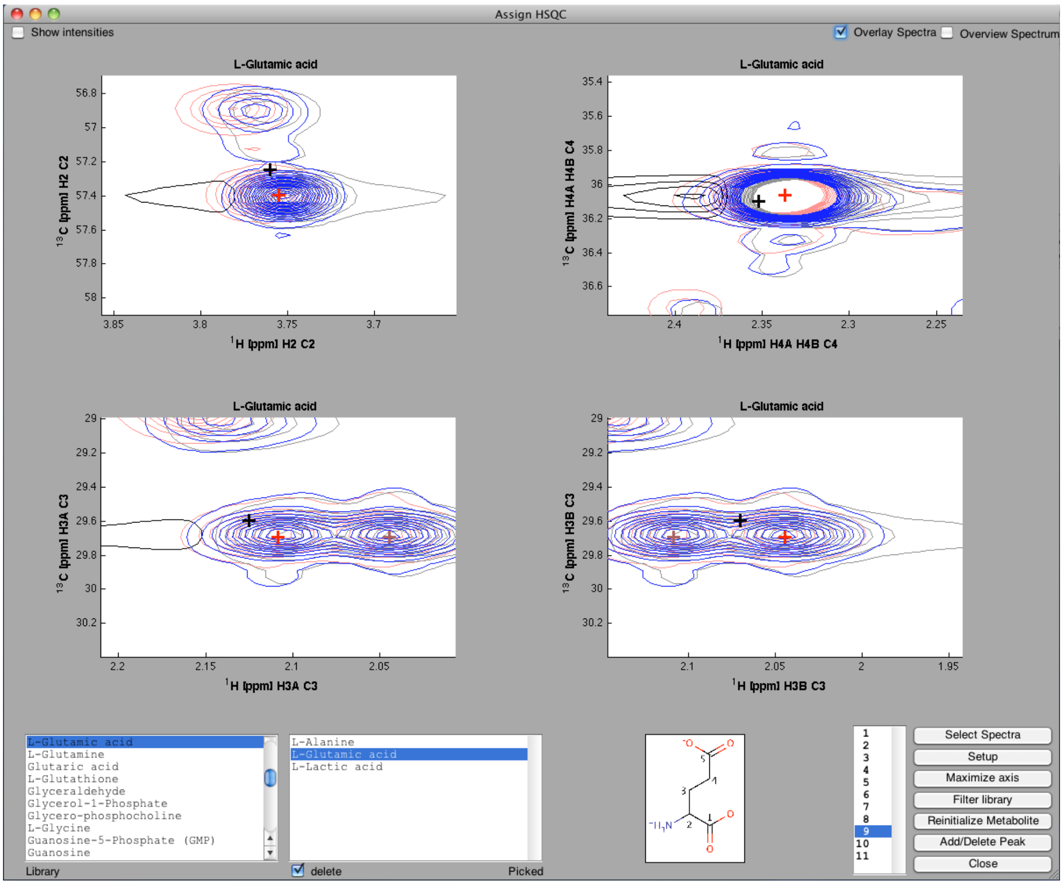

Figure 17: HSQC assignment tool showing the resonances for glutamic acid. The list on the far left in the bottom row shows all metabolites which are available in the spectral library. The listbox right to it shows all the metabolites that have been assigned. The next box on the right shows a molecular structure with the assignment of the nuclei, if available. The listbox on the right allows to select NMR spectra for display.

HSQC assignment tool

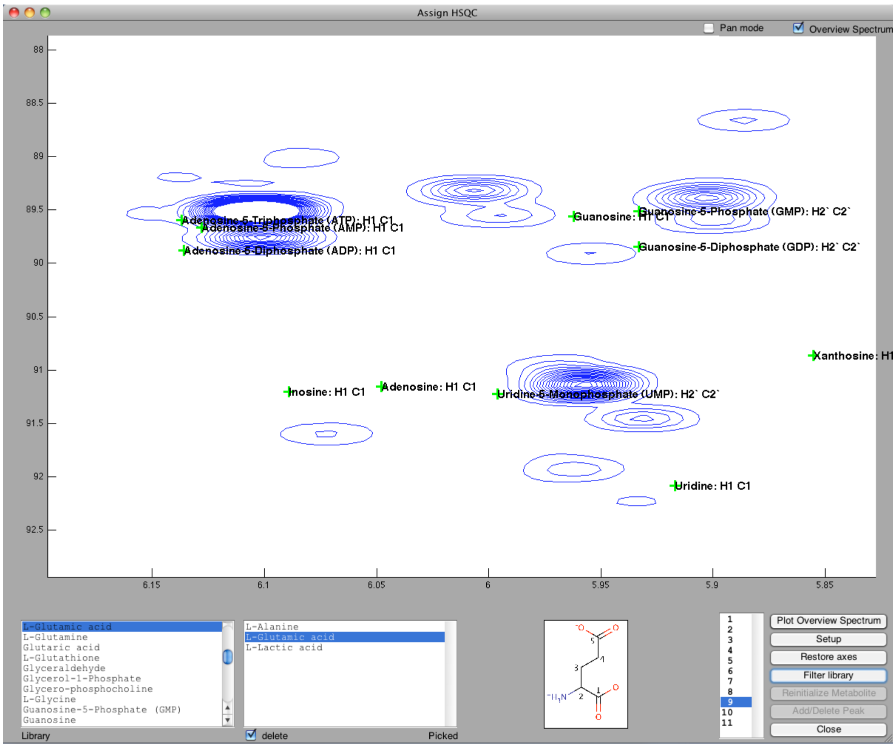

Figure 18: Expanded region of the 2D-^{13}C,^{1}H-HSQC spectrum with possible metabolite assignments from the spectral library.

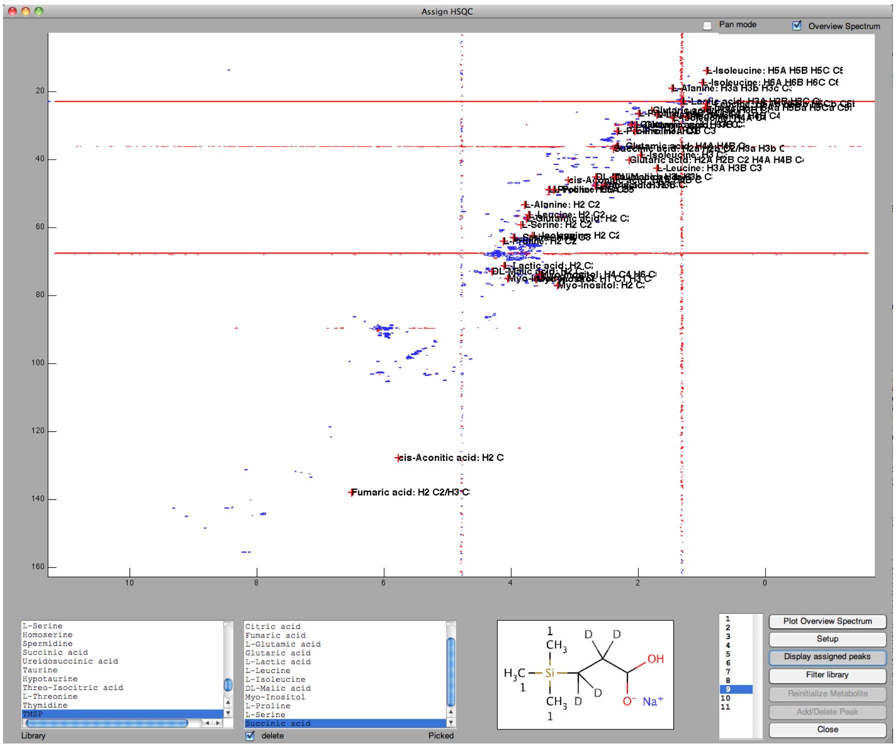

Figure 19: 2D-^{13}C,^{1}H-HSQC spectrum with assigned metabolite resonances.

How to add new metabolites to the library

In this section we show how to add new metabolites to the library. (The instructions imply that the user has write permissions to the NMRLab directories.)

1. Create a text file which will contain the NMR data for the new metabolite (e.g. `glycerate.txt`) The text file should be of the following format:

   ```
   Glyceric acid
   76.1595 (0) 4.1129 (0) 0 0 H2 C2
   66.9266 (0) 3.8158 (0) 0 0 H3A C3
   66.9266 (0) 3.7257 (0) 0 0 H3B C3
   ```

   The line contains the name which will be displayed in the GUI for that metabolite. The first column of the other lines contain the ^{13}C and the third column the ^{1}H chemical shift. The last entry represents the assignment of the peak to a pair of nuclei in the molecule.

2. Create a molecular graphics of the molecule containing the assignment using the same nomenclature as in the text file. The filename should be the same as for the text file (e.g. `glycerate.png`.

3. Run the command `add_metabolites`. This will create matlab scripts which are stored in *$NMRLABPATH*/metlib/metabolites, where *$NMRLABPATH* is the location where NMRLab has been installed.

4. If molecular graphics files exist, copy them to *$NMRLABPATH*/metlib/png.
